# A Machine Learning-Based Mortality Prediction Model for Patients with Chronic Hepatitis C Infection: An Exploratory Study

**DOI:** 10.3390/jcm13102939

**Published:** 2024-05-16

**Authors:** Abdullah M. Al Alawi, Halima H. Al Shuaili, Khalid Al-Naamani, Zakariya Al Naamani, Said A. Al-Busafi

**Affiliations:** 1 Department of Medicine, Sultan Qaboos University Hospital, Muscat 123, Oman; 2 Internal Medicine Program, Oman Medical Specialty Board, Muscat 130, Oman; 3 Department of Medicine, Armed Forces Hospital, Muscat 112, Oman; 4 Department of Medicine, College of Medicine and Health Sciences, Sultan Qaboos University, Muscat 123, Oman

**Keywords:** Chronic hepatitis C, machine learning, prediction model, mortality

## Abstract

**Background:** Chronic hepatitis C (HCV) infection presents global health challenges with significant morbidity and mortality implications. Successfully treating patients with cirrhosis may lead to mortality rates comparable to the general population. This study aims to utilize machine learning techniques to create predictive mortality models for individuals with chronic HCV infections. **Methods:** Data from chronic HCV patients at Sultan Qaboos University Hospital (2009–2017) underwent analysis. Data pre-processing handled missing values and scaled features using Python via Anaconda. Model training involved SelectKBest feature selection and algorithms such as logistic regression, random forest, gradient boosting, and SVM. The evaluation included diverse metrics, with 5-fold cross-validation, ensuring consistent performance assessment. **Results:** A cohort of 702 patients meeting the eligibility criteria, predominantly male, with a median age of 47, was analyzed across a follow-up period of 97.4 months. Survival probabilities at 12, 36, and 120 months were 90.0%, 84.0%, and 73.0%, respectively. Ten key features selected for mortality prediction included hemoglobin levels, alanine aminotransferase, comorbidities, HCV genotype, coinfections, follow-up duration, and treatment response. Machine learning models, including the logistic regression, random forest, gradient boosting, and support vector machine models, showed high discriminatory power, with logistic regression consistently achieving an AUC value of 0.929. Factors associated with increased mortality risk included cardiovascular diseases, coinfections, and failure to achieve a SVR, while lower ALT levels and specific HCV genotypes were linked to better survival outcomes. **Conclusions:** This study presents the use of machine learning models to predict mortality in chronic HCV patients, providing crucial insights for risk assessment and tailored treatments. Further validation and refinement of these models are essential to enhance their clinical utility, optimize patient care, and improve outcomes for individuals with chronic HCV infections.

## 1. Introduction

Hepatitis C virus (HCV) infection is a significant global health concern, affecting over 70 million people worldwide, and contributes to global morbidity and mortality, leading to cirrhosis, hepatocellular carcinoma (HCC), liver transplantation, and liver-related deaths [1]. The 2030 Agenda for Sustainable Development highlights the need to address HCV infections, with world leaders committing to combat this global health issue by 2030. In response, the World Health Organization (WHO) developed the Global Viral Hepatitis Strategy, aiming to reduce deaths by two-thirds and increase treatment rates to 80% [2]. The introduction of direct-acting antiviral (DAA) therapy has transformed HCV clinical care and prompted efforts toward eliminating HCV infections [3,4]. Unfortunately, around 700,000 individuals die annually as a result of HCV-related complications, with many diagnosed only after manifesting symptoms related to either cirrhosis or HCC [5]. Observational studies have primarily focused on determining the advantages of curing HCV, which include a reduced risk of mortality when compared to untreated individuals with chronic HCV infection or those for whom treatment has not succeeded [6,7]. However, the long-term prognosis for successfully treated individuals is still debatable, especially in the era of interferon-free DAA regimens. Multiple studies propose that individuals with cirrhosis who have been effectively treated for HCV show low mortality rates, similar to the general population when adjusted for age, sex, and calendar year [8,9].

Other studies have identified specific factors associated with poor survival among HCV patients, including failure to achieve sustained virological response (SVR), HCC, human immunodeficiency virus (HIV) coinfection, hepatitis B virus (HBV) coinfection, substance abuse, decompensated liver cirrhosis, old age, HCV genotype, diabetes mellitus, and smoking [10,11,12,13,14]. However, the presence of multiple factors, including comorbidities, HIV and HBV coinfection, HCV genotype, and the presence of chronic liver disease, represents unpredictable interactions, leading to various reported HCV survival outcomes [15,16]. Therefore, exploring nonlinear and intricate relationships among different patients and viral characteristics becomes valuable in understanding these complex interactions.

Employing sophisticated statistical tools and machine learning techniques can improve the accuracy of our predictions, such as survival, compared to traditional statistical methods. This improvement is achieved by considering the higher-dimensional and potentially nonlinear influences of different variables, resulting in a broader range of viral and host-related variables included in the analysis [17]. Artificial intelligence (AI) is a rapidly growing field in computer science, finding applications in diverse sectors like e-commerce, media, and finance. While the integration of AI, particularly machine learning (ML), has been relatively slow in the health sciences, it is now garnering interest [18,19]. ML is a subset of AI that emphasizes the learning of algorithms from data. Unlike traditional programing, ML algorithms derive patterns and relationships from subsets of data. There are four main learning methods: supervised (using labeled data), unsupervised (using unlabeled data), semi-supervised (combining labeled and unlabeled data), and reinforcement learning. These methods are useful for different tasks and enable systems to make predictions, solve problems, and make informed decisions [20].

Deep learning, a subset of machine learning, utilizes deep neural networks (DNNs) to process data. DNNs mimic the structure of the cerebral cortex by using multiple layers of interconnected artificial neurons. It consists of nodes that communicate through connections, similar to how cell bodies communicate through axons and dendrites. In biological and artificial networks, synapses between neurons become stronger when their outputs are correlated, shaping the network’s behavior [20,21]. Previous prediction models using AI/ML have primarily focused on predicting the development of HCC, treatment response, and cirrhosis rather than mortality prediction [22,23,24].

The aim of the present study is to use different ML techniques, along with traditional logistic regression, to create and validate models for mortality prediction among chronic HCV-infected patients.

## 2. Materials and Methods

### 2.1. Study Setting and Population

This study used data gathered from a database of patients seen at the adult hepatology clinic at Sultan Qaboos University Hospital (SQUH), a 500-bed tertiary referral hospital specializing in various medical services and featuring specific, specialized medical facilities [25]. All data of patients with chronic HCV referred to the adult hepatology clinic from January 2009 to December 2017, aged 13 years and older, were included. Patients who experienced spontaneous clearance of HCV, patients with missing survival outcomes, and patients who were followed up in alternative healthcare facilities were excluded from this study.

### 2.2. Definitions

The resolution of acute HCV infection was considered when the HCV viral load became undetectable within six months of diagnosis using highly sensitive real-time polymerase chain reaction (PCR) with lower detection limits of 15 IU/mL. Chronic HCV infection was defined as cases with a detectable HCV viral load after six months of infection, confirmed by two positive readings spaced six months apart. Diagnoses of HCC and cirrhosis were made following the guidelines of the European Association for the Study of the Liver (EASL) [26,27].

### 2.3. Features

Relevant data for this study were gathered from patients’ electronic medical records, which included baseline information like demographic data, comorbidities, and relevant laboratory results, including a detailed hematological profile, hepatic and renal function, HCV genotype, and HCV viral load, as well as HBV and HIV coinfection status. Additionally, detailed data related to HCV treatment and outcomes were included. All patients were followed up from their initial encounter at the adult hepatology clinic until death or the follow-up endpoint (April 2022), whichever occurred first.

### 2.4. Outcome

The primary outcome of interest was all-cause mortality during the follow-up period. Additionally, patients with end-stage liver disease or other terminal diagnoses who had been lost to follow-up for three years were considered deceased.

### 2.5. Data Pre-Processing

Before creating the model, the dataset was pre-processed to manage missing values and scale continuous features. For binary features with missing values, these were filled using the mode. Continuous features with missing values were replaced by the median, accommodating their skewed distribution. To ensure consistency and standardize scales for continuous features, we applied the StandardScaler, which standardizes features by subtracting the mean and dividing by the standard deviation. This standardization ensures that the features have zero mean and unit variance [28].

### 2.6. Primary Data Analysis

All data analyses and model development relied on the Anaconda distribution, incorporating the Python programing language along with diverse libraries [29].

The Shapiro–Wilk test from the Scipy Library was utilized to assess the normality of each continuous variable in the dataset. Normally distributed variables were reported as means and standard deviations (SDs), and abnormally distributed variables were reported as medians and interquartile ranges (IQRs). Binary variables were reported as numbers and frequencies. The survival analysis was conducted using the KaplanMeierFitter module from the lifelines library using the Kaplan–Meier method. The median survival time and survival probabilities at specific time points (12, 24, 36, 60, and 120 months) were calculated.

### 2.7. Feature Selection and Model Training

The data were split into training data (80%) and testing data (20%). Feature selection and model training were performed using the sci-kit-learn library in Python. The SelectKBest algorithm was employed to identify relevant features associated with mortality. A SelectKBest object was created with the ANOVA F-value as the score function, and a value of k = 10 was set to select the top 10 features. The SelectKBest object was then fitted to the training data. The get support method was used to retrieve the selected feature. The final models for logistic regression, random forest, gradient boosting, and support vector machine (SVM) were initialized and trained using the selected features, and feature coefficients, importance, and contributions were reported for each feature in the final models.

### 2.8. Model Evaluations

The performance metrics for each model were calculated, including the area under the curve (AUC), accuracy, precision, recall, and F1 score. This allows for a comprehensive evaluation of the performance of each model in terms of its predictive ability. The model’s performance was also evaluated at different time points using a filtered dataset, with time points of interest (12, 24, 36, 60, and 120 months) used to filter the data. The filtered dataset was then split into training and testing sets, and the models were trained on the training set. The probabilities of the positive class were predicted on the testing set, and the AUC was calculated.

### 2.9. Model Cross-Validation

Cross-validations were performed using 5-fold cross-validation for each of the trained models: logistic regression, random forest, gradient boosting, and support vector machine (SVM), with an array of 5 scores for each fold. The cross-validation scores for each model were reported using the area under the receiver operating characteristic curve (ROC AUC), providing an overview of the model’s performance across the 5 folds.

## 3. Results

A total of 702 patients met the inclusion criteria. There were 477 (67.9%) men, and the average age was 47 (IQR: 33–59) years old. The median follow-up duration was 97.4 months (61.0–131.2). Detailed baseline characteristics and relevant clinical laboratory findings are presented in Table 1.

**Figure 1:**
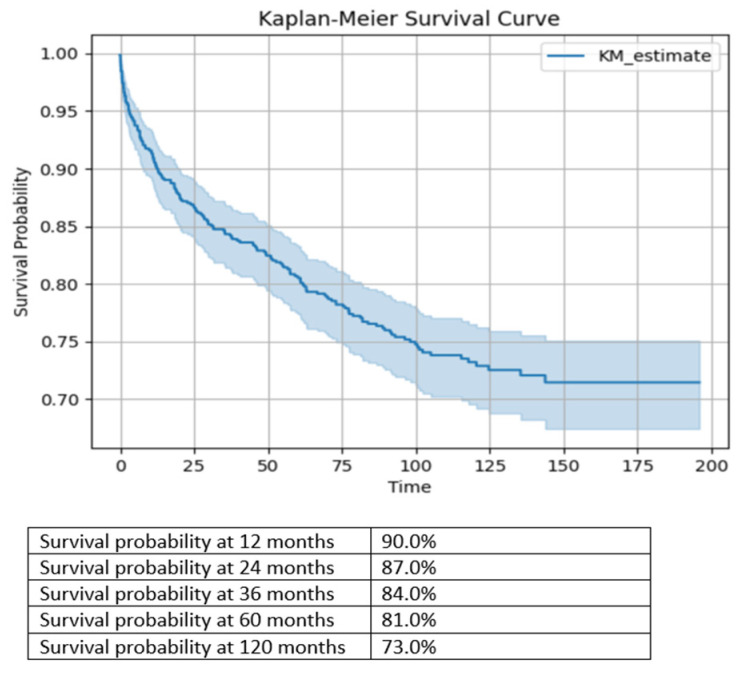
Kaplan–Meier survival curve for patients with chronic HCV infections.

The survival time for patients diagnosed with chronic HCV infections from the index encounter at the adult hepatology clinic until death or the final follow-up date (April 2022) is shown in Figure 1. The survival probabilities were 90.0%, 84.0%, and 73.0% at 12, 36, and 120 months, respectively.

The SelectKBest function from the scikit-learn library was used to select the top 10 features based on the ANOVA F-value. These features, namely hemoglobin level (Hb), alanine aminotransferase (ALT), alpha-fetoprotein, hypertension, cardiovascular system diseases, HCV genotype 1, HCV genotype 3, coinfection with HBV virus, follow-up duration, and SVR, were used for model training and evaluation. Higher hemoglobin levels, the presence of cardiovascular system diseases, HBV virus coinfection, longer follow-up duration, and failure to achieve a SVR were associated with an increased likelihood of mortality in patients with chronic HCV infections. On the other hand, lower levels of alanine aminotransferase, the absence of hypertension, and HCV genotype 3 were associated with improved survival outcomes.

The performance of the different machine learning models in predicting the mortality outcome was evaluated using several performance metrics, as presented in Table 2. The logistic regression, random forest, gradient boosting, and support vector machine models achieved AUC values of 0.926, 0.897, 0.919, and 0.907, respectively.

Cross-validation using five folds resulted in AUC scores ranging from approximately 0.972 to 1.000 for logistic regression models. The random forest model demonstrated variations in performance across different folds, with the cross-validation AUC scores ranging from approximately 0.633 to 1.000. Similarly, the gradient boosting model showed variations in performance across different folds, with the cross-validation AUC scores ranging from approximately 0.270 to 1.000. In contrast, the support vector machine model demonstrated consistent performance across the different folds, with the cross-validation AUC scores ranging from approximately 0.915 to 0.996, as shown in Table 3.

The predictive ability of machine learning models for mortality outcomes over time was assessed at different time points (12, 24, 36, 60, and 120 months). The logistic regression model consistently achieved an AUC of 0.929 at each time point, indicating its stable performance over time, as shown in Table 4.

The random forest model demonstrated varying performance across the different time points, with its AUC values ranging from approximately 0.908 to 0.915. Similarly, the gradient boosting model showed consistent performance, with its AUC values ranging from approximately 0.918 to 0.919 across the time points.

## 4. Discussion

This study presents a novel mortality prediction model using machine learning for patients with chronic HCV infections. The median follow-up duration was approximately 97.4 months, allowing for a significant observation period to analyze mortality outcomes. The prediction models achieved high accuracy levels in predicting mortality at different time points.

The survival probabilities at specific time points, calculated using the Kaplan–Meier method, indicate the long-term prognosis of patients with chronic HCV infections. At 12, 36, and 120 months, the survival probabilities were 90.0%, 84.0%, and 73.0%, respectively. Survival rates can vary in chronic HCV patients, depending on the study population. For instance, a study from the USA reported a 5-year survival rate of 82%, which is consistent with our findings [30]. Another study from South Korea observed high overall survival rates in HCV patients without cirrhosis, reaching 99.7% at 3 years and 96% at 5 years, similar to the general population [30,31].

In a large meta-analysis of 31 studies, including 33,360 patients, it was found that achieving a SVR after HCV treatment significantly reduces the risk of death compared to unsuccessful therapy across different populations. The risk of all-cause mortality decreases by approximately 50% in the general population, 74% in cirrhotic patients, and 79% in coinfected patients [32]. This demonstrates a significant survival benefit, even in patients with cirrhosis and HIV coinfection. These findings highlight the relatively high survival rate among patients treated for CHC. However, it is important to note that various factors, such as treatment advances, adherence to therapy, and patient characteristics, may influence these survival probabilities [32].

The SelectKBest algorithm was utilized to identify the top 10 features associated with mortality. Raised alpha-fetoprotein, failure to achieve a SVR, and coinfection with the HBV were associated with high mortality, consistent with the findings of previous studies [32,33]. In addition, lower levels of ALT, the absence of hypertension, and the presence of HCV genotype 3 were associated with improved survival outcomes in patients with chronic HCV infections. However, factors such as age, HCC, liver cirrhosis, other non-hepatic malignancies, and liver transplants were not among the top 10 features that impacted patient survival. This highlights the comprehensive nature of machine learning models in predicting outcomes by considering the complex interactions of different patients and viral factors beyond those specifically related to liver diseases [34].

The performance of different machine learning models in predicting mortality outcomes was evaluated using various performance metrics, including the AUC, accuracy, precision, recall, and F1 score. The logistic regression model displayed the highest AUC value of 0.926, indicating its excellent discriminatory ability in distinguishing between survivors and non-survivors. Logistic regression models have been widely used in medical research and are known for their simplicity and interpretability [35]. In this study, the logistic regression model showed promising performance in predicting mortality outcomes, suggesting its potential utility as a predictive tool for clinicians.

The random forest, gradient boosting, and support vector machine (SVM) models also showed good predictive performances, with AUC values of 0.897, 0.919, and 0.907, respectively. These models utilize complex algorithms that capture nonlinear relationships and interactions between different variables, improving prediction accuracy [36,37]. The consistent performance of these models across multiple performance metrics suggests their robustness in predicting mortality outcomes in patients with chronic HCV infections.

Cross-validation was performed to assess the generalizability of these models. The logistic regression model showed high cross-validation AUC scores, ranging from approximately 0.972 to 1.000. This demonstrates the model’s stability and reliability across different folds. The random forest and gradient boosting models showed variations in their performance across different folds, indicating potential sensitivity to changes in the training dataset. In contrast, the support vector machine model demonstrated consistent performance across different folds, boosting confidence in its predictive ability. Employing cross-validation is an important step to ensure that the machine learning models can be moderated against excessive optimism and potential biases arising from hyperparameter tuning and algorithm selection [38].

Additionally, the predictive performance of these models was evaluated at different time points to assess their ability to predict mortality outcomes over time. The logistic regression model consistently achieved an AUC of 0.929, indicating its stable performance. The random forest model showed varying performance across different time points, with its AUC values ranging from approximately 0.908 to 0.915. The gradient boosting model demonstrated consistent performance, with its AUC values ranging from approximately 0.918 to 0.919. Similarly, the support vector machine model consistently achieved an AUC of approximately 0.925 across all time points. These findings suggest that these models have the potential to predict mortality outcomes with reasonable accuracy at different stages of the disease.

It is important to note that the predictive performance of these models should be interpreted cautiously. Although the models showed good discrimination and predictive abilities, they are not without limitations. The models were developed using data from a single center and a specific patient population, which may limit their generalizability to other populations. Furthermore, the models were trained on retrospective data, and the retrospective nature of the study may introduce biases and limit causal inference. Prospective validation studies are needed to confirm the predictive accuracy of these models in diverse patient populations.

In addition, the models may not capture all relevant factors contributing to mortality outcomes in patients with chronic HCV infections. There may be other unmeasured variables that influence long-term prognoses, such as lifestyle factors, socioeconomic status, and access to healthcare. Including these factors in future studies may improve the predictive accuracy of these models.

Despite these limitations, the findings of this study provide valuable insights into the prediction of mortality outcomes in patients with chronic HCV infections. The use of machine learning algorithms and feature selection techniques offers a promising approach to identifying high-risk patients who may benefit from early interventions and targeted treatment strategies. The models can assist clinicians in risk stratification and personalized management, ultimately leading to improved patient outcomes.

Future research should focus on the external validation of these models in independent cohorts to assess their generalizability and applicability in different healthcare settings. Additionally, efforts should be made to incorporate additional variables and risk factors that may contribute to mortality outcomes. Long-term prospective studies are warranted to further refine and optimize predictive models for mortality in patients with chronic HCV infections.

## 5. Conclusions

The present study demonstrates the potential of ML models in predicting mortality outcomes in patients with chronic HCV infections. The logistic regression, random forest, gradient boosting, and support vector machine models showed good performances in discriminating between survivors and non-survivors. These models highlight the importance of factors such as hemoglobin level, ALT level, alpha-fetoprotein level, comorbidities, HCV genotype, coinfection with the HBV, and SVRs in predicting mortality outcomes. Further research and validation are needed to confirm the utility of these models and refine their predictive accuracy. Implementing these predictive models in clinical practice has the potential to improve risk stratification and individualized treatment strategies for patients with chronic HCV infections, ultimately leading to better patient outcomes and healthcare resource allocation.

## Figures and Tables

**Table 1:** Demographic, clinical, and laboratory characteristics of the study population.

Characteristics	Total *n* = 702
Age (years)	47 (33–59)
Men	477 (67.9%)
Weight (kg)	67 (57–76)
Height (cm)	160 (156–167)
Body Mass Index (BMI; kg/m^2^)	25.3 (22.2–29.1)
History of drug abuse	182 (25.9%)
History of alcohol	151 (21.5%)
Smoking	95 (13.5%)
Liver cirrhosis	244 (34.8%)
Decompensated liver cirrhosis	93 (13.2%)
Diabetes mellitus	198 (28.2%)
Liver transplant	23 (3.3%)
HIV coinfection	12 (1.7%)
Other malignancy	41 (5.8%)
Hypertension	91 (13.0%)
Sickle cell disease or thalassemia	148 (21.1%)
Cardiovascular disease	97(13.8%)
Chronic kidney disease	76 (10.8%)
Hepatitis B coinfection	253 (36.0%)
Sustained virological response (SVR)	320 (45.6%)
HCV genotype 1	290 (41.3%)
HCV genotype 2	27 (3.8%)
HCV genotype 3	221 (31.5%)
HCV genotype 4	81 (11.5%)
HCV genotype 5	15 (2.1%)
Ultrasound elastography (N/m^2^)	7.5
White cell count × 109/L	6 (4.5–8.6)
Absolute neutrophil count × 109/L	2.8 (1.8–4.3)
Hemoglobin (g/dL)	12.4 (10.4–14.0)
Platelets × 109/L	222 (143–300)
International normalized ratio (INR)	1.04 (1.0–1.2)
Alanine aminotransferase (ALT) U/L	50.5 (29.0–86.0)
Aspartate aminotransferase (AST) U/L	49.0 (29.0–84.0)
Alkaline phosphatase (ALP) U/:	87.0 (69.0–121.0)
Bilirubin (umol/L)	11.0 (7.0–22.0)
Albumin (g/L)	40.0 (33.0–44.0)
Serum creatinine level (umol/L)	66.0 (52.0–81.8)
Serum chloride (mmol/L)	3.9 (3.8–4.0)
alpha-fetoprotein levels (AFP) (ng/mL)	3.0 (2.0–4.0)
Ferritin level (ng/mL)	217 (214.0–217.8)
HbA1c %	5.8 (5.6–5.9)
HCV load (IU/L)	590,220.0 (132,550.0–2,481,070)
Hepatocellular carcinoma	71 (10.1%)
Follow-up duration (months)	97.4 (61.0–131.2)

**Table 2:** Metrics highlighting the performance of each model in terms of its discriminative ability (AUC), overall accuracy, precision (ability to identify positive cases correctly), recall (ability to capture all true positives), and F1 score (a harmonic mean of precision and recall).

Model	AUC	Accuracy	Precision	Recall	F1 Score
Logistic regression	0.926	0.887	0.868	0.750	0.805
Random forest	0.897	0.915	1.000	0.727	0.842
Gradient boosting	0.919	0.894	0.939	0.705	0.805
Support vector machine	0.907	0.872	0.933	0.636	0.757

**Table 3:** Cross-validation ROC AUC scores for machine learning models.

Model	Fold 1	Fold 2	Fold 3	Fold 4	Fold 5
Logistic regression	0.993	0.990	1.000	0.999	0.972
Random forest	0.825	1.000	1.000	0.754	0.633
Gradient boosting	0.588	1.000	0.995	0.270	0.690
Support vector machine	0.996	0.992	0.999	0.987	0.915

**Table 4:** AUC values for machine learning models at 12, 24, 36, 60, and 120 months.

Model	12 Months	24 Months	36 Months	60 Months	120 Months
Logistic regression	0.929	0.929	0.929	0.929	0.929
Random forest	0.915	0.900	0.919	0.910	0.907
Gradient boosting	0.918	0.919	0.918	0.919	0.919
Support vector machine	0.925	0.925	0.925	0.925	0.925

## Data Availability

All data generated or analyzed during this study are included in this article. Further inquiries can be directed to the corresponding author.

## References

[1] Di Marco L., La Mantia C., Di Marco V. (2022). Hepatitis C: Standard of Treatment and What to Do for Global Elimination. Viruses.

[2] Lombardi A., Mondelli M.U., ESCMID Study Group for Viral Hepatitis (ESGVH) (2019). Hepatitis C: Is eradication possible?. Liver Int..

[3] Martinello M., Solomon S.S., Terrault N.A., Dore G.J. (2023). Hepatitis C. Lancet.

[4] Manns M.P., Maasoumy B. (2022). Breakthroughs in hepatitis C research: From discovery to cure. Nat. Rev. Gastroenterol. Hepatol..

[5] Shahnazarian V., Ramai D., Reddy M., Mohanty S. (2018). Hepatitis C virus genotype 3: Clinical features, current and emerging viral inhibitors, future challenges. Ann. Gastroenterol..

[6] Butt A.A., Yan P., Shaikh O.S., Lo Re V., Abou-Samra A.B., Sherman K.E. (2020). Treatment of HCV reduces viral hepatitis-associated liver-related mortality in patients: An ERCHIVES study. J. Hepatol..

[7] Butt A.A., Wang X., Moore C.G. (2009). Effect of hepatitis C virus and its treatment on survival. Hepatology.

[8] McDonald S.A., Innes H.A., Aspinall E., Hayes P.C., Alavi M., Valerio H., Goldberg D.J., Hutchinson S.J. (2017). Prognosis of 1169 hepatitis C chronically infected patients with decompensated cirrhosis in the predirect-acting antiviral era. J. Viral Hepat.

[9] Bruno S., Di Marco V., Iavarone M., Roffi L., Crosignani A., Calvaruso V., Aghemo A., Cabibbo G., Viganò M., Boccaccio V. (2016). Survival of patients with HCV cirrhosis and sustained virologic response is similar to the general population. J. Hepatol..

[10] Choi G.H., Jang E.S., Kim Y.S., Lee Y.J., Kim I.H., Cho S.B., Lee H.C., Jang J.W., Ki M., Choi H.Y. (2022). Hepatocellular carcinoma, decompensation, and mortality based on hepatitis C treatment: A prospective cohort study. World J. Gastroenterol..

[11] Song A.T.W., Sobesky R., Vinaixa C., Dumortier J., Radenne S., Durand F., Calmus Y., Rousseau G., Latournerie M., Feray C. (2016). Predictive factors for survival and score application in liver retransplantation for hepatitis C recurrence. World J. Gastroenterol..

[12] Fujiyama S., Akuta N., Sezaki H., Kobayashi M., Kawamura Y., Hosaka T., Kobayashi M., Saitoh S., Suzuki F., Suzuki Y. (2021). Mortality rates and risk factors in 1412 Japanese patients with decompensated hepatitis C virus-related cirrhosis: A retrospective long-term cohort study. BMC Gastroenterol..

[13] Butt Z.A., Wong S., Rossi C., Binka M., Wong J., Yu A., Darvishian M., Alvarez M., Chapinal N., Mckee G. (2020). Concurrent Hepatitis C and B Virus and Human Immunodeficiency Virus Infections Are Associated With Higher Mortality Risk Illustrating the Impact of Syndemics on Health Outcomes. Open Forum Infect. Dis..

[14] Park H.K., Lee S.S., Bin Im C., Im C., Cha R.R., Kim W.S., Cho H.C., Lee J.M., Kim H.J., Kim T.H. (2019). Hepatitis C virus genotype affects survival in patients with hepatocellular carcinoma. BMC Cancer.

[15] Terrault N.A., Roland M.E., Schiano T., Dove L., Wong M.T., Poordad F., Ragni M.V., Barin B., Simon D., Olthoff K.M. (2012). Outcomes of liver transplant recipients with hepatitis C and human immunodeficiency virus coinfection. Liver Transpl..

[16] Sterling R.K., Sulkowski M.S. (2004). Hepatitis C virus in the setting of HIV or hepatitis B virus coinfection. Semin. Liver Dis..

[17] Angraal S., Mortazavi B.J., Gupta A., Khera R., Ahmad T., Desai N.R., Jacoby D.L., Masoudi F.A., Spertus J.A., Krumholz H.M. (2020). Machine Learning Prediction of Mortality and Hospitalization in Heart Failure With Preserved Ejection Fraction. JACC Heart Fail..

[18] Aung Y.Y.M., Wong D.C.S., Ting D.S.W. (2021). The promise of artificial intelligence: A review of the opportunities and challenges of artificial intelligence in healthcare. Br. Med. Bull..

[19] Kashoub M., Al Abdali M., Al Shibli E., Al Hamrashdi H., Al Busaidi S., Al Rawahi M., Al Alawi A. (2023). Artificial Intelligence in Medicine: A Double-edged Sword or a Pandora’s Box?. Oman Med. J..

[20] Choi R.Y., Coyner A.S., Kalpathy-Cramer J., Chiang M.F., Campbell J.P. (2020). Introduction to Machine Learning, Neural Networks, and Deep Learning. Transl. Vis. Sci. Technol..

[21] Bhat M., Rabindranath M., Chara B.S., Simonetto D.A. (2023). Artificial intelligence, machine learning, and deep learning in liver transplantation. J. Hepatol..

[22] Zou Y., Yue M., Jia L., Wang Y., Chen H., Zhang A., Xia X., Liu W., Yu R., Yang S. (2023). Accurate prediction of HCC risk after SVR in patients with hepatitis C cirrhosis based on longitudinal data. BMC Cancer.

[23] Colak C., Kucukakcali Z., Akbulut S. (2023). Artificial intelligence-based prediction of molecular and genetic markers for hepatitis C-related hepatocellular carcinoma. Ann. Med. Surg..

[24] Minami T., Sato M., Toyoda H., Yasuda S., Yamada T., Nakatsuka T., Enooku K., Nakagawa H., Fujinaga H., Izumiya M. (2023). Machine learning for individualized prediction of hepatocellular carcinoma development after the eradication of hepatitis C virus with antivirals. J. Hepatol..

[25] Al-Yarabi A., Al Balushi H., Al Hatmi K., Al Yahyaie R., Al Alawi A.M., Al Zeedy K., Al Farhan H. (2023). Inappropriate Hospital Stay of Patients Admitted Under Care of General Medicine Units: A retrospective study. Sultan Qaboos Univ. Med. J..

[26] European Association for the Study of the Liver (2018). EASL Clinical Practice Guidelines for the management of patients with decompensated cirrhosis. J. Hepatol..

[27] European Association for the Study of the Liver (2018). EASL Clinical Practice Guidelines: Management of hepatocellular carcinoma. J. Hepatol..

[28] Pfob A., Lu S.C., Sidey-Gibbons C. (2022). Machine learning in medicine: A practical introduction to techniques for data pre-processing, hyperparameter tuning, and model comparison. BMC Med. Res. Methodol..

[29] Mikkelsen L., Moesgaard K., Hegnauer M., Lopez A.D. (2020). ANACONDA: A new tool to improve mortality and cause of death data. BMC Med..

[30] van der Meer A.J., Wedemeyer H., Feld J.J., Dufour J.-F., Zeuzem S., Hansen B.E., Janssen H.L.A. (2014). Life expectancy in patients with chronic HCV infection and cirrhosis compared with a general population. JAMA.

[31] Pak S.C., Alastal Y., Khan Z., Darr U. (2017). Viral Hepatitis in South Korea. Euroasian J. Hepatogastroenterol..

[32] Simmons B., Saleem J., Heath K., Cooke G.S., Hill A. (2015). Long-Term Treatment Outcomes of Patients Infected With Hepatitis C Virus: A Systematic Review and Meta-analysis of the Survival Benefit of Achieving a Sustained Virological Response. Clin. Infect. Dis..

[33] Minami Y., Aoki T., Chishina H., Takita M., Hagiwara S., Ida H., Ueshima K., Nishida N., Kudo M. (2022). Prognostic Factors for Overall Survival in Patients with HCV-Related HCC Undergoing Molecular Targeted Therapies: Beyond a Sustained Virological Response. Cancers.

[34] Pettit R.W., Fullem R., Cheng C., Amos C.I. (2021). Artificial intelligence, machine learning, and deep learning for clinical outcome prediction. Emerg. Top. Life Sci..

[35] Schober P., Vetter T.R. (2021). Logistic Regression in Medical Research. Anesth. Analg..

[36] Siemers F.M., Bajorath J. (2023). Differences in learning characteristics between support vector machine and random forest models for compound classification revealed by Shapley value analysis. Sci. Rep..

[37] Speiser J.L. (2021). A random forest method with feature selection for developing medical prediction models with clustered and longitudinal data. J. Biomed. Inform..

[38] Bradshaw T.J., Huemann Z., Hu J., Rahmim A. (2023). A Guide to Cross-Validation for Artificial Intelligence in Medical Imaging. Radiol. Artif. Intell..

